# Disentangling the contributions of cerebrovascular-related white matter integrity markers to cognitive aging

**DOI:** 10.1016/j.cccb.2025.100395

**Published:** 2025-09-12

**Authors:** Elmira Agah, Sarah T. Farias, David K. Johnson, Charles DeCarli, Pauline Maillard

**Affiliations:** a Department of Neurology, University of California, Davis, CA, United States; b Imaging of Dementia and Aging (IDeA) Laboratory and Center for Neurosciences, Davis, CA, United States

**Keywords:** White matter integrity, Diffusion tensor imaging, Cognition, Longitudinal, Aging

## Abstract

**Objective:**

To determine the independent and joint associations of five markers of white matter integrity, including white matter hyperintensities (WMH), extracellular free water (FW), fractional anisotropy (FA), peak width of skeletonized mean diffusivity (PSMD), and Diffusion tensor image analysis along the perivascular space index (ALPS) on cognitive performance and its trajectory in cognitively diverse individuals.

**Methods:**

574 participants from the University of California, Davis Alzheimer’s Disease Research Center (UCD ADRC) longitudinal cohort, aged 77 ± 7 years received yearly comprehensive clinical evaluations and a baseline MRI exam. Baseline MRI measures, including WMH, FW, FA, PSMD, and ALPS, were computed for each individual and used as independent variables to explain baseline and change in episodic memory (EM) and executive function (EF) using linear regression with stepwise adjustment. Bayesian Model Averaging (BMA) was then applied to derive robust estimates of each marker’s contribution to cognition and its longitudinal trajectory, accounting for their joint inclusion in the same model.

**Results:**

Analyses showed that higher baseline WMH, FW, and PSMD, as well as lower FA and ALPS, were significantly associated with poorer cognitive performance (*p* < 0.05). These associations remained robust after adjusting for relevant covariates—including age, sex, education, hypertension, diabetes, and hippocampal volume—except for FA and ALPS, which were no longer associated with EM (p > 0.05). Higher baseline WMH, FW, and PSMD, and lower FA, were also significantly associated with annual decline in EF and EM, whereas ALPS showed no association with cognitive change (p > 0.05). After covariate adjustment, these associations remained significant, except for PSMD and FA which were no longer significantly associated with EM trajectory. Joint modeling using BMA identified FW and WMH as the most likely contributors to both baseline performance and change in EF and EM, with posterior inclusion probabilities exceeding 50 %.

**Conclusions:**

This study identified both cross-sectional and longitudinal associations between five markers of white matter integrity and cognitive performance and decline. Using BMA—a method designed to disentangle the specific contribution of each marker while accounting for multicollinearity—we found that, among the five markers, FW and WMH emerged as the most probable candidates to explain the course of cognitive decline in EM and EF.

## Highlights

• Free water (FW) and white matter hyperintensities (WMH) were the strongest predictors of both baseline cognitive performance and longitudinal decline, outperforming fractional anisotropy (FA), peak width of skeletonized mean diffusivity (PSMD), and the diffusion tensor imaging along the perivascular space index (ALPS).
• FW was more strongly linked to executive function, while WMH showed the strongest and most consistent association with episodic memory.
• Bayesian Model Averaging (BMA) identified FW and WMH as the most probable contributors to cognitive performance and trajectory, while other markers displayed minimal or inconsistent effects on cognitive outcomes.

## Introduction

Cognitive function is a critical determinant of health and well-being, particularly with advancing age [1]. With the global increase in life expectancy, the prevalence of cognitive dysfunction and neurodegenerative disorders is expected to rise significantly in the coming decades [2]. Cognitive decline poses challenges that affect individuals, their families, and the healthcare system [3,4]. It often leads to higher medical costs, decreased independence, and a substantial reduction in quality of life [3,4]. Identifying reliable biomarkers for the early detection of brain changes that may signal future cognitive decline is becoming increasingly important. These biomarkers can enhance our understanding of brain aging and cognitive impairment and may help guide strategies to slow the process or manage its consequences.

Neuroimaging provides a unique window into brain structure and function and has become an essential tool for identifying early markers of neurodegeneration. While most studies focus on markers of gray matter degeneration, measures of white matter integrity are also important, as white matter damage plays a critical role in the development and progression of cognitive impairment [5]. White matter hyperintensities (WMH), visible as bright lesions on FLAIR MRI sequences, represent chronic white matter damage and are commonly associated with small vessel disease [6], although they are also associated with AD degeneration [7]. In addition to WMH, diffusion tensor imaging (DTI) provides sensitive measures of microstructural health in white matter [8]. These include extracellular free water (FW), a marker of neuroinflammation or tissue degeneration; fractional anisotropy (FA), which reflects the directionality of water diffusion and white matter organization; and the peak width of skeletonized mean diffusivity (PSMD), a summary measure of microstructural disruption linked to small vessel disease [[8], [9], [10]]. More recently, the analysis along the perivascular space (ALPS) index has emerged as a potential marker of glymphatic dysfunction, which may be involved in the pathophysiology of neurodegenerative processes [11,12].

Previous studies have shown that WMH are associated with cognitive impairment, particularly in executive function and processing speed [13]. WMH are considered a core marker of small vessel disease and tend to accumulate with age and vascular risk [14]. DTI also provides additional insights into white matter structure. FA generally decreases with aging and has been associated with cognitive decline in several domains, including memory and executive function. FW reflects the extracellular water content and is believed to capture early microstructural damage resulting from neuroinflammation or tissue degeneration [15]. Elevated FW has been reported in aging individuals and in those with mild cognitive impairment [16,17]. PSMD is a summary measure that captures the variability of mean diffusivity across the white matter skeleton [18]. Higher PSMD values have been associated with poorer cognitive performance, particularly in processing speed and executive function, and have been increasingly used as a marker of small vessel disease [18,19]. Lower ALPS values may indicate impaired brain clearance mechanisms [20], which is thought to contribute to neurodegenerative diseases such as Alzheimer’s disease [21]. Although ALPS is a promising marker, its utility in predicting cognition remains uncertain.

While each of these imaging markers has been individually studied in relation to cognition, it remains unclear which ones are most informative for detecting early brain changes that may lead to cognitive decline. Few studies have directly compared them in the same model to evaluate their relative contributions to cognitive function [19]. This is especially important because these markers may reflect different underlying mechanisms, such as vascular injury, microstructural disruption, neuroinflammation, or impaired glymphatic clearance. Understanding which markers are more strongly associated with cognitive performance and its change over time may help prioritize their use in future studies and potentially in clinical assessments.

This study aims to determine the independent and joint associations of five markers of white matter integrity—including WMH, FW, FA, PSMD, and the ALPS index—with baseline cognitive performance and cognitive trajectory in a cognitively diverse sample.

## Methods

### Study population

Individuals included in this study are a subgroup of University of California, Davis Alzheimer’s Disease Research Center (UCD ADRC) longitudinal cohort. Approximately 74 % of the participants were recruited through community based recruitment protocols designed to enhance racial and ethnic diversity and the spectrum of cognitive dysfunction with an emphasis on normal cognition and MCI [22]. The other 26 % of the sample initially sought a clinical evaluation at the UCD ARDC. Regardless of recruitment source, exclusion criteria included unstable major medical illness, major primary psychiatric disorder, and substance abuse or dependence in the last five years.

The MRI sample included 574 individuals who received a standardized MRI scan of the brain including a diffusion tensor imaging (DTI) acquisition and a clinical examination. Mean ± standard deviation (SD) time difference between baseline MRI and baseline cognitive assessment was 0.05 ± 0.24 years. Yearly cognitive assessments were completed for all subjects. Mean ± SD time difference between baseline and the latest follow-up cognitive assessment available was 4.9 ± 3.5 years (see Supplemental Figure S1 for individual follow-up examination statistics). The presence or absence of hypertension or diabetes at baseline was assessed for everyone based on thorough review of the participant’s medical history, medical records, and medications brought into the clinic at the time of the evaluation.

### Standard protocol approvals, registrations, and patient consents

The Institutional Review Boards at all participating institutions approved this study, and individuals or their legal representatives gave written informed consent.

### Clinical diagnosis and cognitive outcomes

For all participants, determination of clinical syndrome was made at baseline. The diagnosis of AD was made according to the National Institute of Neurological and Communication Disorders and Stroke/Alzheimer’s Disease and Related Disorders Association criteria [23]. Mild cognitive impairment (MCI) was diagnosed according to current consensus criteria [24]. Individuals were declared cognitively unimpaired (CU) if there was no clinically significant cognitive impairment, defined as performance on any clinical cognitive tests greater than 1.5 standard deviations below age and education adjusted means.

All participants received a comprehensive clinical evaluation and neuropsychological testing from a standardized test battery on a yearly basis. The primary cognitive outcome measures in this study were the executive function (EF) and episodic memory (EM) measures from the Spanish and English Neuropsychological Assessment Scales (SENAS), which is derived using item response theory (IRT) and psychometrically matched across scales and across English and Spanish versions [[25], [26], [27]], lack appreciable floor or ceiling effects, have linear measurement properties across a broad ability range, and have equivalent reliability and sensitivity. As a result, the EF and EM scores are standardized and directly comparable across participants and across studies that employ SENAS, as has been demonstrated in multiple published cohorts. The measures are continuously distributed across this cohort, including individuals diagnosed as CU, MCI, and AD, thus justifying the inclusion of all three diagnostic groups into unified models of WMH effects on cognition. Mixed model random effects regression analyses were used to model the annual rate of change of the cognitive outcome measures, i.e. annual change in executive function and in episodic memory (ΔEF and ΔEM) [28].

### Image processing

Structural MRI scans were obtained at the UCD MRI research center on a 3T Siemens Magnetom Trio Syngo System and a 1.5T GE Signa Genesis system. Acquired images included a T1-weighted volumetric magnetization-prepared rapid gradient echo and a fluid-attenuated inversion recovery (FLAIR) scan. Detailed description of image acquisition has been provided previously [29,30].

Indicators of white matter integrity include white matter hyperintensities (WMH) measured with a FLAIR, as well as extracellular free water (FW), fractional anisotropy (FA), peak width of skeletonized mean diffusivity (PSMD), and Diffusion tensor image analysis along the perivascular space index (ALPS) captured through diffusion tensor imaging (DTI). Quantification of WMH was achieved with a previously described algorithm [[31], [32], [33], [34]]. using a Bayesian approach estimated from FLAIR images. Methodology for generating FW, FA, PSMD and ALPS measures has been previously described [35,36]. Briefly, the model used to compute FW and FA considers two co‐existing compartments per voxel: one compartment is a FW compartment, which models isotropic diffusion with a diffusion coefficient of water at body temperature (37 °C) fixed to 3 × 10^−3^ mm^2^/s and a second compartment, which accounts for all other molecules, i.e., all intra- and extracellular molecules that are hindered or restricted by physical barriers like axonal membranes and myelin [15,37]. Global measures of mean FW and FA were computed by averaging values within white matter (WM) voxels for each participant [38]. Elevated FW is often interpreted as a marker of neuroinflammation or tissue degeneration and lower FA suggests microstructural damage or demyelination. PSMD is a summary marker of small vessel disease burden derived from the distribution of MD values along the white matter skeleton. Larger PSMD values reflect greater white matter microstructural disruption. ALPS is a diffusion-based measure thought to reflect glymphatic system function by quantifying diffusivity along perivascular spaces. Lower ALPS values suggest impaired interstitial fluid clearance. A more detailed description of the methods used to generate the five markers can be found in the Supplemental Method.

Other MRI variables included hippocampal volume and total intracranial volume (TCV). Hippocampal segmentation employed a harmonized atlas [[39], [40], [41]] based diffeomorphic approach [34] with atlas fusion utilizing MALF [42,43] and intensity-based label refinement. TCV segmentation and quantification were performed using a convolutional neural network method, as described previously [44].

WMH volumes, FW and PSMD were log-transformed to normalize population variance. All WM integrity measures, including WMH, FW, FA, PSMD and ALPS, as well as hippocampus volume were residualized by TCV to adjust for differences due to head size and standardized for facilitating regression coefficient comparison.

### Statistical analyses

#### Independent association between WM integrity markers and cognition

Our first goal was to investigate the independent effect of WM integrity marker, including WMH, FW, FA, PSMD and ALPS, on cognitive performance and its trajectory. To test this hypothesis, we performed a stepwise adjustment approach: for each WM integrity marker, we first used separate linear regression with baseline EM or EF score as the dependent variable and the WM integrity marker as the independent variable (Model M1). We then adjusted Model M1 for age, sex, education and TCV (Model M2). In a third model, we additionally adjusted for vascular risk factors including history of diabetes and hypertension (Model M3). This model aimed to estimate the added contribution of the imaging marker above vascular risk factors on executive functions and episodic memory. Finally, in a fourth model, the model was also adjusted by hippocampus volume to also evaluate the contribution of the imaging marker above neurodegeneration pathology (Model M4). To investigate the independent role of the WM integrity markers on cognitive trajectory, we repeated similar models but using ΔEM and ΔEF as the dependent variable.

While these models provide interpretable estimates of association strength between each of the WM integrity marker, including WMH, FW, FA, PSMD, ALPS and cognition, they do not however decipher the specificity of each marker in the presence of other markers.

#### Joint evaluation of WM integrity marker effects on cognition

To address this question, and given the substantial collinearity among imaging measures, we applied Bayesian Model Averaging (BMA) [45] using the *bicreg* function from the BMA R package. BMA considers all possible combinations of predictors and computes a model-averaged coefficient (posterior mean) and its posterior standard deviation, weighted by each model’s posterior probability given the data. This approach mitigates model selection bias and instability due to multicollinearity, offering a more robust estimate of each marker’s contribution. We also report the posterior inclusion probability (PIP) for each predictor, which reflects the probability that a given variable is included in the true underlying model, given the data and the set of candidate models. It is a direct measure of the importance or relevance of that variable in explaining the outcome. A high PIP indicates consistent inclusion across the best-fitting models but does not imply a larger effect size compared to variables with lower PIPs. We used BMA rather than alternative variable importance methods such as random forest because it yields interpretable regression coefficients and PIPs, and directly accounts for multicollinearity among predictors. This approach aligns with our aim to quantify the independent contribution of each WM integrity marker, rather than to optimize predictive accuracy. Predictors with PIP > 50 % and credible intervals excluding zero were interpreted as reliably associated with EF, EM, ΔEF and ΔEM.

#### Replication in non-demented individuals

To ensure that associations between MRI measures and episodic memory and executive functions were not overestimated by inclusion of demented individuals in the analysis, we replicated all analyses described above including only CU and MCI individuals.

## Results

### Baseline subjects’ characteristics

As compared to MCI, CU participants were younger (*p* = 0.0015, see Table 1), had lower WMH (<0.001), FW (<0.001), PSMD (<0.001) and TCV (<0.001) and higher FA (<0.00), ALPS (<0.001) and hippocampus volume (<0.001). As compared to AD participants, CU participants were younger (*p* = 0.0015), had lower FW (<0.001) and PSMD (<0.001) and higher FA (<0.00) and hippocampus volume (<0.001). MCI participants did not differ from AD participants, except that hippocampus volume was larger in MCI (<0.001, see Table 1).

**Table 1:** Baseline subjects’ characteristics.

	Total	CU	MCI	AD	P
N	574	359	156	59	
Age (years)	75.76 ± 7 [49.02; 95.35]	74.95 ± 6.59 [60.13; 95.35]	77.06 ± 7.02 [54.6; 92.03]	77.3 ± 8.54 [49.02; 91]	<0.001^ξω^
Sex	229; 39.9	131; 36.5	71; 45.5	27; 45.8	0.099
Hypertension	372; 68.5	229; 67.2	105; 72.4	38; 66.7	0.49
Diabetes	175; 32.2	119; 34.9	42; 29	14; 24.6	0.18
Education (years)	14.37 ± 4.01 [0; 20]	14.35 ± 4.02 [0; 20]	14.69 ± 4.1 [0; 20]	13.58 ± 3.66 [5; 20]	0.19
WMH volume (cc)	9.56 ± 12.42 [0.07; 133.94]	7.65 ± 10.22 [0.07; 64.1]	13.57 ± 15.91 [0.35; 133.94]	10.48 ± 11.63 [0.76; 65.46]	<0.001^ω^
FW	-0.01 ± 0.19 [-0.57; 0.74]	-0.06 ± 0.17 [-0.57; 0.4]	0.07 ± 0.18 [-0.39; 0.63]	0.07 ± 0.22 [-0.37; 0.74]	<0.001^ξω^
FA	0 ± 0.03 [-0.13; 0.16]	0 ± 0.03 [-0.05; 0.16]	-0.01 ± 0.02 [-0.07; 0.05]	-0.01 ± 0.03 [-0.13; 0.05]	<0.001^ξω^
PSMD	-0.01 ± 0.26 [-0.88; 1.42]	-0.07 ± 0.24 [-0.88; 1.42]	0.08 ± 0.24 [-0.53; 0.89]	0.1 ± 0.32 [-0.47; 0.97]	<0.001^ξω^
ALPS	0 ± 0.18 [-0.62; 0.52]	0.01 ± 0.18 [-0.52; 0.51]	-0.03 ± 0.19 [-0.62; 0.52]	-0.03 ± 0.17 [-0.41; 0.34]	<0.001^ω^
Hippocampus (cc)	5.94 ± 0.85 [3.59; 8.29]	6.23 ± 0.71 [3.92; 8.29]	5.62 ± 0.81 [3.72; 7.65]	5.06 ± 0.86 [3.59; 7.47]	<0.001^τξω^
TCV (cc)	1191.92 ± 124.99 [884.98; 1516.08]	1178.43 ± 119.22 [884.98; 1499.94]	1218.46 ± 131.59 [972.52; 1516.08]	1202.9 ± 130.72 [921; 1497.4]	<0.001^ω^

Values are mean ± standard deviation [range] for continuous variables and N ( %) for categorical variables. p-values reflect group differences using *t*-tests (continuous) or Chi-square tests (categorical). Significant (p<0.05) differences between CU and AD participants (ξ), between CU and MCI participants (ω), and between MCI and AD participants (τ), are shown by ξ, ω and τ, respectively, using *post hoc* analyses. FW, FA, PSMD, and ALPS are standardized residuals adjusted for TCV.

Abbreviations: AD = Alzheimer’s disease; ALPS = analysis along the perivascular space; CU = cognitively unimpaired; FA = fractional anisotropy; FW = free water; MCI = mild cognitive impairment; PSMD = peak width of skeletonized mean diffusivity; TCV = total cranial volume.

### Independent association between WM integrity markers and cognition

#### Baseline cognition

Cross-sectional analyses revealed that both EF and EM were strongly associated with each of the WM integrity markers, with increased WMH, FW and PSMD and decreased FA and ALPS being associated with worse cognitive outcomes (p<0.05, see Table 2). Adding age, sex, education and TCV did not alter these associations, except for ALPS and EM (*p* = 0.19). Associations also remained significant when adding vascular risk factors and hippocampus volume, except for EM and FA (*p* = 0.07). When comparing effect sizes in the final models, i.e. including all adjustment covariates, FW and WMH appeared to be the strongest predictors of EF and EM respectively (see Table 2).

**Table 2:** Linear regression/ Cross-sectional cognitive outcomes.

	M1		M2		M3		M4	
	EF	EM	EF	EM	EF	EM	EF	EM
WMH	-0.27 (0.041); <0.001	-0.29 (0.04); <0.001	-0.19 (0.04); <0.001	-0.23 (0.042); <0.001	-0.19 (0.042); <0.001	-0.23 (0.045); <0.001	-0.170 (0.04); <0.001	-0.190 (0.039); <0.001
FW	-0.28 (0.04); <0.001	-0.3 (0.04); <0.001	-0.27 (0.043); <0.001	-0.23 (0.046); <0.001	-0.26 (0.045); <0.001	-0.24 (0.049); <0.001	-0.220 (0.044); <0.001	-0.170 (0.043); <0.001
FA	0.18 (0.041); <0.001	0.13 (0.041); 0.0016	0.12 (0.037); 0.0018	0.084 (0.04); 0.034	0.11 (0.039); 0.0051	0.077 (0.042); 0.07	0.100 (0.038); 0.008	0.058 (0.037); 0.120
PSMD	-0.25 (0.041); <0.001	-0.25 (0.041); <0.001	-0.21 (0.04); <0.001	-0.17 (0.044); <0.001	-0.2 (0.042); <0.001	-0.18 (0.045); <0.001	-0.160 (0.041); <0.001	-0.100 (0.04); 0.012
ALPS	0.095 (0.042); 0.023	0.089 (0.042); 0.033	0.13 (0.038); <0.001	0.054 (0.041); 0.19	0.13 (0.039); 0.0011	0.062 (0.042); 0.15	0.100 (0.038); 0.009	0.010 (0.037); 0.800

Values are β (SE); p-value. Model M1: univariate association. Model M2: M1 + Age + Sex + Education + TCV. Model M3: M1 + Age + Sex + Education + TCV + Hypertension + Diabetes. Model M4: M1 + Age + Sex + Education + TCV + Hypertension + Diabetes + Hippocampus volume.

Abbreviations: ALPS = analysis along the perivascular space; EF = executive function; EM = episodic memory; FA = fractional anisotropy; FW = free water; PSMD = peak width of skeletonized mean diffusivity; TCV = total cranial volume.

#### Cognitive trajectory

Associations between WM integrity markers and annual change in executive function and episodic memory are summarized in Table 3. The distribution of individual annual change in EF and EM in relation to baseline scores is shown in Supplemental Figures S2 and S3. Accelerated decline in EF and EM were found to be strongly associated with increased baseline WMH, FW and PSMD and decreased baseline FA (p<0.001) but not with ALPS (*p* = 0.21 and *p* = 0.60 respectively). These significant associations remained unchanged when adding age, sex, education and TCV, as well as vascular risk factors (p<0.05, see Table 2). Associations also remained significant when adjusting the models for hippocampus volume, except between PSMD and EM (*p* = 0.18). Again, when comparing effect sizes in the final models, FW and WMH were found to be the strongest predictors of EF and EM trajectory respectively (see Table 2).

**Table 3:** Linear regression/ Longitudinal cognitive outcomes.

	M1		M2		M3		M4	
	ΔEF	ΔEM	ΔEF	ΔEM	ΔEF	ΔEM	ΔEF	ΔEM
WMH	-0.29 (0.041); <0.001	-0.26 (0.041); <0.001	-0.26 (0.045); <0.001	-0.23 (0.046); <0.001	-0.28 (0.047); <0.001	-0.24 (0.048); <0.001	-0.25 (0.044); <0.001	-0.22 (0.046); <0.001
FW	-0.3 (0.04); <0.001	-0.24 (0.041); <0.001	-0.3 (0.049); <0.001	-0.19 (0.05); <0.001	-0.32 (0.051); <0.001	-0.21 (0.053); <0.001	-0.26 (0.048); <0.001	-0.16 (0.051); 0.0015
FA	0.16 (0.041); <0.001	0.14 (0.041); <0.001	0.12 (0.042); 0.0041	0.11 (0.043); 0.0086	0.13 (0.045); 0.003	0.11 (0.045); 0.019	0.12 (0.042); 0.0047	0.094 (0.043); 0.031
PSMD	-0.23 (0.042); <0.001	-0.17 (0.042); <0.001	-0.18 (0.047); <0.001	-0.1 (0.047); 0.036	-0.19 (0.048); <0.001	-0.12 (0.049); 0.014	-0.12 (0.046); 0.0069	-0.063 (0.047); 0.18
ALPS	0.07 (0.042); 0.095	0.035 (0.042); 0.41	0.045 (0.044); 0.3	-0.013 (0.044); 0.76	0.047 (0.045); 0.3	0.0043 (0.046); 0.92	0.0033 (0.042); 0.94	-0.032 (0.044); 0.46

Values are β (SE); p-value. Model M1: univariate association. Model M2: M1 + Age + Sex + Education + TCV. Model M3: M1 + Age + Sex + Education + TCV + Hypertension + Diabetes. Model M4: M1 + Age + Sex + Education + TCV + Hypertension + Diabetes + Hippocampus volume.

Abbreviations: ALPS = analysis along the perivascular space; EF = executive function; EM = episodic memory; FA = fractional anisotropy; FW = free water; PSMD = peak width of skeletonized mean diffusivity; TCV = total cranial volume; Δ= annual change.

### Joint evaluation of WM integrity marker effects on cognition using BMA

Compared to independent association models, which suggested several significant associations, the BMA framework aims at identifying a more conservative subset of markers with high inclusion probabilities, highlighting those that contribute independently of collinear effects (see Table 3, Table 4).

**Table 4:** Bayesian model averaging with baseline cognitive outcomes.

	Executive Function			Episodic Memory		
	PIP	Estimate	SE	PIP	Estimate	SE
FW	91.5	−0.197	0.044	13.6	−0.011	0.045
FA	0.9	0.000	0.043	3.6	0.001	0.038
PSMD	9.5	−0.012	0.044	10.9	−0.007	0.039
WMH	42.3	−0.049	0.043	100	−0.193	0.038
ALPS	14.9	0.011	0.039	3	0.001	0.036
Hippocampus	100	0.243	0.037	100	0.462	0.035
Age	2.7	−0.001	0.044	0	0.000	NA
Sex	100	0.210	0.042	100	0.246	0.036
Education	100	0.438	0.037	100	0.240	0.035
Hypertension	2.7	−0.001	0.037	0	0.000	NA
Diabetes	64.9	−0.066	0.037	0	0.000	NA
TCV	65	0.086	0.048	9.1	−0.007	0.046

PIP: posterior inclusion probability. Estimate: model-averaged β coefficient; SE: posterior standard deviation.

Abbreviations: ALPS = analysis along the perivascular space; EF = executive function; EM = episodic memory; FA = fractional anisotropy; FW = free water; PSMD = peak width of skeletonized mean diffusivity; TCV = total cranial volume.

#### Baseline cognition

The BMA analysis revealed that, among the 5 markers of WM integrity, (i.e WMH, FW, FA, PSMD, and ALPS), FW was the most probable candidate to explain performances in EF (posterior inclusion probability [PIP]: 91.5 %, β=-0.197, see Table 4). For the other markers, PIP values ranged from 0.9 % for FA (β = 0.00) to 42.3 % for WMH (β = –0.049), indicating that these biomarkers are relatively weak contributors to the model explaining executive function. WMH was found to be the most probable candidate to explain performances in EM (PI*p* = 100, β=-0.193, see Table 5). Other WM integrity markers exhibited very low PIP (PIPs< 14 %, see Table 4). For both EF and EM, hippocampal volume, sex, and education each had a PIP of 100 %, highlighting their strong relevance in explaining performance in both cognitive domains.

**Table 5:** Bayesian model averaging with longitudinal cognitive outcomes.

	Annual change in Executive Function			Annual change in Episodic Memory		
	PIP	Estimate	SE	PIP	Estimate	SE
FW	66.4	−0.105	0.055	3.5	−0.002	0.053
FA	3.4	0.002	0.042	0	0.000	NA
PSMD	5	0.001	0.053	0	0.000	NA
WMH	96.6	−0.187	0.045	100	−0.213	0.043
ALPS	2.4	−0.001	0.042	3.7	−0.001	0.041
Hippocampus	100	0.359	0.040	100	0.321	0.041
Age	20.7	0.022	0.048	0	0.000	NA
Sex	100	0.145	0.039	79.4	0.097	0.042
Education	96.2	0.130	0.039	6.1	0.003	0.040
Hypertension	0	0.000	NA	3.6	0.001	0.041
Diabetes	0	0.000	NA	5.9	0.003	0.041
TCV	0	0.000	NA	10.8	−0.008	0.047

PIP: posterior inclusion probability. Estimate: model-averaged β coefficient; SE: posterior standard deviation. Outcomes represent annual change in EF and EM.

Abbreviations: ALPS = analysis along the perivascular space; EF = executive function; EM = episodic memory; FA = fractional anisotropy; FW = free water; PSMD = peak width of skeletonized mean diffusivity; TCV = total cranial volume.

#### Cognitive trajectory

BMA analyses on longitudinal change in EF and EM performance evidenced that both FW and WMH were probable candidates to explain trajectory in EF (PI*p* = 66.4, β = -0.105 for FW; PI*p* = 96.6, β = -0.187 for WMH, see Table 5). WMH was also found to be the most probable candidate to explain trajectory in EM (PI*p* = 100, β = -0.213 for WMH). Again, for both EF and EM trajectory, hippocampus volume, sex and education were found to have a PIP of 100, indicating the relevance of each of these variables in explaining the annual change in EF and EM performance.

### Additional analyses

Excluding individuals with an advanced stage of cognitive impairment did not significantly alter associations between markers of WM integrity and cognitive performance described above (See Supplemental Data).

## Discussion

In this cognitively diverse cohort of older adults, we evaluated the independent and joint contributions of five white matter integrity markers—WMH, FW, FA, PSMD, and ALPS—in relation to cognitive performance and trajectory. The main findings of this study can be summarized as follows: 1) all five WM integrity markers, when examined independently, were significantly associated with cognitive performance, with greater WMH, FW, and PSMD, and lower FA and ALPS values linked to worse cognitive outcomes, 2) All markers except ALPS were significantly associated with annual cognitive decline rate, 3) FW and WMH emerged as the strongest and most consistent predictors of both cognitive performance and trajectory, 4) when using Bayesian Model Averaging (BMA) to account for multicollinearity, FW was the most probable marker explaining EF, whereas WMH was the most probable marker explaining EM, 5) regarding the cognitive trajectory, both FW and WMH were identified as the most likely markers for the decline of EF, while WMH alone emerged as the strongest predictor of EM decline.

Findings from the present study align with previous studies that show microstructural WM damage is associated with cognitive decline [46,47]. Altered DTI measures, particularly decreased FA values, have consistently been linked to poor cognitive function in prior studies [48]. Longitudinal studies have shown that FA values decrease in cognitively normal individuals with aging, potentially reflecting ongoing microstructural degradation secondary to age-related health factors, including vascular risk burden and accumulating comorbid conditions [49]. However, this decline is typically less extensive and relatively more widespread compared to individuals with Alzheimer’s disease (AD), where patterns are typically more localized [49,50]. These findings may indicate that microstructural changes may precede the development of visible tissue damage as WMH on conventional imaging and serve as an early marker of cognitive impairment and dementia.

In the present study, FW and WMH were the strongest predictors of cognitive function and cognitive trajectory across all modeling approaches, including univariate, fully adjusted, and BMA models. This consistency may highlight their potential utility as reliable imaging biomarkers in studies of cognitive aging. These two imaging markers may represent different but complementary aspects of white matter pathology. FW measures elevated fluid levels in brain white matter, potentially indicating acute or ongoing microstructural changes such as neuroinflammation, interstitial edema, or axonal degeneration [51]. These changes may occur before or alongside more noticeable structural injuries and can even be present in areas that seem normal on conventional MRI, prior to the development of new WMH [52,53]. A recent study conducted in a comparable cohort of older adults with a range of vascular risk factors and varying levels of cognitive function, further supports the role of FW as an early marker of white matter damage [54]. The authors found that elevated plasma levels of placental growth factor (PlGF), a biomarker of vascular dysfunction, were associated with higher white matter FW [54]. FW partially mediated the association between PlGF and cognitive status and fully mediated the association between PlGF and WMH volume [54]. Together, these findings suggest FW may serve as a critical link between vascular burden and both structural and functional brain changes relevant to cognitive aging.

Interestingly, in BMA models, FW had a higher PIP than WMH for cross-sectional EF (91.5 % vs 42.3 %), while WMH was more predictive of EF decline (PIP = 96.6 % vs 66.4). This pattern suggests that FW is more sensitive to current functional impairment, while WMH has a stronger association with disease progression. Alternatively, WMH may associate more strongly with progression as it is a marker of white matter of more severe white matter injury [52]. It is important to note that our analysis was not designed to formally test whether FW or WMH is statistically better at predicting a specific cognitive domain; instead, differences in PIPs indicate that each marker may be more relevant for certain domains when considered alongside other related predictors. We propose that the combination of elevated FW and WMH may indicate an active phase of pathology [52], which should be further explored in future longitudinal studies.

PSMD, a relatively novel DTI-derived metric, also showed significant associations with cognitive outcomes, though its predictive value was weaker compared to FW and WMH. PSMD reflects the variability in mean diffusivity (MD) across major WM tracts and has been suggested as a sensitive marker of diffuse small vessel disease burden [18,55]. The association between PSMD and cognitive performance, especially EF, observed in this study aligns with prior research indicating that PSMD is closely linked to processing speed and overall cognition [18,19]. Although PSMD was significantly associated with cognitive performance, its ability to predict cognitive decline was more limited compared to FW and WMH in BMA models. This may suggest that PSMD captures general microstructural damage but is less sensitive to ongoing WM changes that underlie longitudinal cognitive decline.

Unlike the other markers, ALPS did not show meaningful associations with cognitive outcomes. Although lower ALPS was associated with worse cross-sectional EF scores, it was not significantly associated with cognitive trajectory and had consistently low PIPs in BMA models. DTI-ALPS is an innovative MRI technique that evaluates water diffusivity in perivascular spaces and is utilized to examine the health and functionality of glymphatic system of the brain [56]. Lower ALPS has been previously shown to be associated with cognitive impairment [20]. In contrast to our findings, in another cohort of older adults with varying cognitive status, higher ALPS index levels were associated with less decline in the global cognitive scores over time [20]. Moreover, individuals who converted from MCI to AD had significantly lower ALPS values at baseline compared to non-converters [20]. Glymphatic system dysfunction is just one of several pathological processes contributing to cognitive decline [57]. This dysfunction can lead to extracellular fluid accumulation, which is also reflected by increased FW. A recent study involving four independent cohorts further clarified this relationship, showing that FW partially mediates the link between ALPS and executive function [58]. Unlike ALPS, FW offers a more integrative signal and can serve as a more sensitive and comprehensive marker of early white matter injury and cognitive aging.

EF is one of the first cognitive domains to decline with aging [59]. A common trend we observed in our analyses was that markers of WM integrity had a greater correlation with EF than with EM. This pattern likely reflects fundamental neuroanatomical differences between these cognitive domains. Executive function is heavily dependent on frontal-subcortical circuits, which are particularly susceptible to white matter damage from small vessel disease [60,61]. In contrast, EM is primarily supported by medial temporal lobe (MTL) structures, including the hippocampus [62], which are not optimally captured by the WM markers evaluated in this study. Another interesting result of this study was the consistent superiority of WMH as a predictor of EM, both cross-sectionally and longitudinally (PIP = 100 % in both models) which may reflect poor memory encoding that reduced performance [63]. In contrast, other imaging markers showed minimal contributions to the prediction of EM. This pattern suggests that the cumulative burden of small vessel disease, as captured by WMH, may impair key WM pathways supporting episodic memory. These findings highlight the need to select biomarkers aligned with specific cognitive domains when researching brain aging and the risk of dementia.

One of the main strengths of this study is the thorough evaluation of multiple WM markers within a cognitively diverse, community-based cohort. By using BMA models, we were able to account for multicollinearity and identify the most probable predictors of cognitive performance and trajectory. Additionally, modeling cognitive trajectories over time strengthened our ability to evaluate predictors of decline. However, several limitations should be acknowledged. First, the observational, cross-sectional design limits interpretability and precludes causal inference. Although cognitive trajectories were modeled longitudinally, WM imaging markers were assessed at a single time point, which prevents evaluation of their temporal dynamics. Additionally, long-term cognitive changes were not examined, which may limit our understanding of disease progression. A potential limitation of longitudinal cognitive studies is the influence of practice effects from repeated testing. However, we conducted mixed-effects models including visit order to account for retest gains and the findings remained unchanged (data not shown). Second, WM integrity markers were calculated as global averages across the brain. This method may overlook region-specific effects that are closely related to certain cognitive domains or early pathological processes. Third, while the cognitive diversity of the cohort enhances the generalizability of our findings, it could also introduce confounding factors. Variability in diagnosis, disease stage, comorbidities, and lifestyle factors that were not fully captured or adjusted for in our models may influence the results. Lastly, we did not examine sex- or ethnicity-specific associations, which could offer important insights given known disparities in WM aging and dementia risk. Such stratified or interaction-based analyses were beyond the scope of the present study but represent an important direction for future research.

**Fig. 1:**
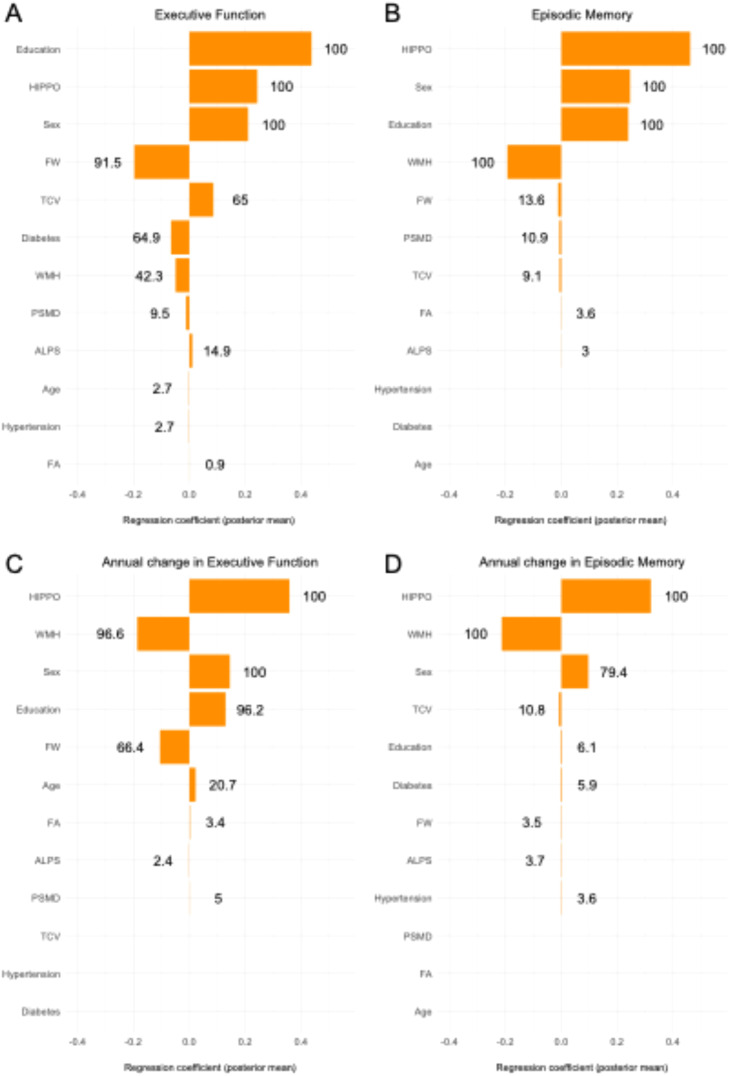
Bayesian Model Averaging posterior mean and posterior inclusion probability.

This study provides a comprehensive comparison of five markers of white matter integrity that reflect complementary mechanisms, including vascular injury (WMH, PSMD), microstructural integrity (FA, FW), and glymphatic clearance (ALPS), in relation to cognitive performance and decline in a cognitively diverse cohort of older adults. FW and WMH consistently emerged as the most valuable markers of cross-sectional and longitudinal executive function and episodic memory, highlighting their utility as key imaging biomarkers of cognitive aging. While other markers, such as PSMD and FA, provided evidence for the role of microstructural damage in cognitive decline, their predictive value was less significant. ALPS, despite its promise as a glymphatic system marker, showed limited relevance in this context. From a translational standpoint, FW and WMH could be incorporated into clinical workflows, as both can be obtained from MRI sequences that are increasingly available in routine practice. WMH quantification is already used in some clinical contexts, and FW can be derived from diffusion-weighted imaging. However, consistent acquisition protocols, reliable automated processing, and normative reference data are needed before these markers can be implemented for early detection or risk stratification Fig. 1.

Taken together, the findings from the present study emphasize the importance of selecting domain-sensitive biomarkers and support the potential of combining multiple imaging metrics, particularly FW and WMH, to improve the early diagnosis and monitoring of cognitive decline.

Summary of Bayesian Model Averaging models on baseline executive function (A) and episodic memory (B) and change in executive function (C) and episodic memory (D). Number next to orange bar indicates the marginal inclusion probability of the variable (see Methods). FW: free water content; FA: fractional anisotropy; PSMD: peak width of skeletonized mean diffusivity (PSMD); ALPS: diffusion tensor image analysis along the perivascular space index; TCV: total cranial volume.

## CRediT authorship contribution statement

**Elmira Agah:** Writing – original draft, Formal analysis. **Sarah T. Farias:** Writing – review & editing, Methodology. **David K. Johnson:** Writing – review & editing, Methodology. **Charles DeCarli:** Writing – review & editing, Methodology, Funding acquisition, Conceptualization. **Pauline Maillard:** Writing – review & editing, Supervision, Methodology, Formal analysis.

## Declaration of competing interest

The authors declare that they have no known competing financial interests or personal relationships that could have appeared to influence the work reported in this paper.

## Appendix Supplementary materials

Image, application 1
