# Supplementary material for: Disentangling the contributions of cerebrovascular-related white matter integrity markers to cognitive aging
Source: Cereb Circ Cogn Behav. 2025 Sep 12;9:100395. doi: 10.1016/j.cccb.2025.100395 (PMC12464545; doi:10.1016/j.cccb.2025.100395)
Supplement: Supplementary file 1 [file mmc1.docx]

# Supplemental Data

**Disentangling the Contributions of Cerebrovascular-Related White Matter Integrity Markers to Cognitive Aging**

Elmira Agah^1,2^, MD, Sarah T. Farias^1^, PhD; David K. Johnson^1^, PhD, Charles DeCarli^1,2^, MD, Pauline Maillard^1,2^, PhD

1 Department of Neurology, University of California, Davis, CA

2 Imaging of Dementia and Aging (IDeA) Laboratory and Center for Neurosciences, Davis, CA

# Supplemental Method

## Image processing

### White matter hyperintensities (WMH)

The algorithm uses high resolution 3D T1-weighted image, a raw FLAIR image and a binary brain mask. The algorithm returns a four-component (cerebral spinal fluid (CSF), gray matter, white matter, and WMH) gray scale segmented image volume in the native space of the 3D T1-weighted volume along with a segmented mask of WMH. A step-by-step analytic plan has been described previously^1^. It includes 1) linear co-registration of 3D T1-weighted image to the FLAIR image, 2) removal of non-brain elements from the FLAIR image using 3D T1-weighted brain mask, 3) image intensity normalization of the FLAIR image, 4) non-linear warping of 3D T1-weighted brain image to a minimal deformation template^2^, 5) non-linear deformation of the FLAIR volume to the atlas using the registration parameters for the 3D T1-weighted volume, 6) application of Bayesian segmentation to both 3D T1-weighted and the FLAIR volumes, 7) creation of four-tissue segmentation volume, 8) reverse transformation of three-tissue segmented volume into 3D T1-weighted native space, 9) reverse transformation of WMH segmented volume into FLAIR native space, 10) reverse transformation of four-tissue segmented volume into 3D T1-weighted native space, 11) output of these volumes into the directory from which the program is launched.

DTI dataset were preprocessed using FSL software tools^3^ including correction for eddy current-induced distortions and participant's head movements. The brain was masked using the BET tool and fractional anisotropy (FA), mean diffusivity (MD), axial diffusivity (AD), and radial diffusivity (RD) maps generated using DTIFIT (FMRIB software library; <http://fsl.fmrib.ox.ac.uk/fsl/fslwiki>).

### Free water (FW) and fractional anisotropy (FA)

The model considers two co‐existing compartments per voxel: one compartment is a free‐water compartment, which models isotropic diffusion with a diffusion coefficient of water at body temperature (37 °C) fixed to 3 × 10^−3^ mm^2^/s^4^. The free‐water fraction is expected to predominantly highlight water molecules in the extracellular space. The second compartment is the tissue compartment, which accounts for all other molecules, i.e., all intra- and extracellular molecules that are hindered or restricted by tissue membranes^5^. The script contains the following steps: 1) the tissue compartment is modeled by a diffusion tensor characterizing the “tissue” molecules, as well as the fractional volume of the free‐water compartment in each voxel, resulting in the FW fraction map, 2) the individual FA map obtained from DTIFIT is linearly and non-linearly registered to the standard FSL FA template space (FMRIB 1-mm FA template) using linear and nonlinear transformations, 3) the resulting transformation parameters are applied to the FW map, 4) a WM mask is defined by thresholding the FSL FA template at a value of 0.3 to reduce cerebrospinal fluid (CSF) partial volume contamination^6^, 5) an overall measure of mean FW and mean FA are computed by superimposing the WM mask onto the individual coregistered FW fraction and Fa map respectively and averaging values within these WM voxels.

### Peak Width of Skeletonized Mean Diffusivity (PSMD)

The PSMD method has been previously described^7^. Briefly, it requires FA, MD, RD, and AD maps. The script procedure includes the following steps: 1) the FA volume is linearly and non-linearly registered to the standard space FMRIB FSL 1-mm FA template; 2) a white matter skeleton is created using the standard Tract-based Spatial Statistics (TBSS)^8^ pipeline available in FSL; 3) subject’s FA data is then projected onto the skeleton, which is derived from the standard space template thresholded at an lower-bound FA value of 0.2 to exclude predominantly non-white matter voxels^6^; 4) MD volume is projected onto the mean FA skeleton using the FA-derived projection parameters and further thresholded with a template skeleton mask to reduce CSF partial volume contamination; 5) PSMD is calculated as the difference between the 95th and 5th percentiles of the voxel-based MD values within the subject’s MD skeleton.

### Diffusion tensor image analysis along the perivascular space index (ALPS)

The pipeline for ALPS index calculation was described previously^10^. Briefly, the 4D DTI volume DICOM files were converted to NIFTI files using MRIcroGL GUI. Then, an in-house bash script (http://loft-lab.org/index-5.html) was used to compute the ALPS index using DTI images as input and including FMRIB Software Library (FSL) and MRtrix3 commands. The DTI images underwent pre-processing, including denoising and Gibbs-unringing, susceptibility-induced distortion, eddy currents and movement corrections, then the fractional anisotropy (FA) map and x-, y-, and z-axis diffusivity maps (Dxx, Dyy, Dzz) were generated after Tensor fitting. The FA map was co-registered to the JHU-ICBM-FA template and the transformation matrix was applied to the Dxx, Dyy and Dzz maps. The projection and association fibers at the level of lateral ventricle body were recognized as the superior corona radiata (SCR) and the superior longitudinal fasciculus (SLF) based on the JHU-ICBM-DTI-81-White-Matter Labeled Atlas. The ROIs were defined as spheres with 5 mm diameter and were placed in the areas of bilateral SCR and SLF which applied on all Dxx, Dyy and Dzz maps. The ALPS index was calculated as follows: ALPS index = $\frac{\boldsymbol{Mean (Dxxproj, Dxxassoc)}}{\boldsymbol{Mean (Dyyproj, Dzzassoc)}}$ . The ALPS indexes of the left and right hemispheres were calculated separately. We used the average ALPS index of the bilateral hemisphere in the following statistical analysis. A lower ALPS index indicates a dysfunction of glymphatic clearance function.

Finally, to further remove differences due to scanner type, DTI measures, including FW, FA, PSMD and ALPS, were corrected ComBat, a method developed to adjust for batch effects in microarray expression data^9^ and proved to be robust at reducing machine related differences in MRI data^10-12^.

**Supplemental Table S1** Linear regression/ Cross-sectional cognitive outcomes in non-demented participants

|  | M1 (Univariate) | | M1 + Age +Sex +Education + TCV | | M1 + Age +Sex +Education + TCV + Hypertension + Diabetes | | M1 + Age +Sex +Education + TCV + Hypertension + Diabetes + Hippocampus | |
| --- | --- | --- | --- | --- | --- | --- | --- | --- |
|  | EF | EM | EF | EM | EF | EM | EF | EM |
| WMH | -0.27 (0.043); <0.001 | -0.31 (0.042); <0.001 | -0.21 (0.042); <0.001 | -0.26 (0.044); <0.001 | -0.19 (0.044); <0.001 | -0.25 (0.047); <0.001 | -0.18 (0.044); <0.001 | -0.22 (0.043); <0.001 |
| FW | -0.26 (0.043); <0.001 | -0.3 (0.042); <0.001 | -0.27 (0.045); <0.001 | -0.23 (0.048); <0.001 | -0.26 (0.048); <0.001 | -0.24 (0.051); <0.001 | -0.24 (0.047); <0.001 | -0.18 (0.047); <0.001 |
| FA | 0.2 (0.043); <0.001 | 0.15 (0.044); <0.001 | 0.14 (0.039); <0.001 | 0.11 (0.041); 0.0075 | 0.15 (0.041); <0.001 | 0.11 (0.044); 0.015 | 0.13 (0.041); 0.0012 | 0.077 (0.041); 0.058 |
| PSMD | -0.22 (0.043); <0.001 | -0.25 (0.043); <0.001 | -0.21 (0.043); <0.001 | -0.18 (0.045); <0.001 | -0.19 (0.045); <0.001 | -0.19 (0.047); <0.001 | -0.16 (0.044); <0.001 | -0.13 (0.044); 0.0041 |
| ALPS | 0.093 (0.044); 0.036 | 0.086 (0.044); 0.05 | 0.13 (0.04); 0.0016 | 0.054 (0.042); 0.2 | 0.13 (0.041); 0.0021 | 0.057 (0.044); 0.2 | 0.11 (0.041); 0.0062 | 0.02 (0.041); 0.62 |

Values indicate linear regression statistics: beta (SE); p. Model M1 corresponds to the univariate association model.

FW: free water content; FA: fractional anisotropy; PSMD: peak width of skeletonized mean diffusivity (PSMD); ALPS: diffusion tensor image analysis along the perivascular space index; TCV: total cranial volume; EF: executive function; EM: episodic memory.

**Supplemental** **Table S2** Linear regression/ Longitudinal cognitive outcomes in non-demented participants

|  | M1 (Univariate) | | M1 + Age +Sex +Education + TCV | | M1 + Age +Sex +Education + TCV + Hypertension + Diabetes | | M1 + Age +Sex +Education + TCV + Hypertension + Diabetes + Hippocampus | |
| --- | --- | --- | --- | --- | --- | --- | --- | --- |
|  | ΔEF | ΔEM | ΔEF | ΔEM | ΔEF | ΔEM | ΔEF | ΔEM |
| WMH | -0.29 (0.041); <0.001 | -0.26 (0.041); <0.001 | -0.26 (0.045); <0.001 | -0.23 (0.046); <0.001 | -0.28 (0.047); <0.001 | -0.24 (0.048); <0.001 | -0.25 (0.044); <0.001 | -0.22 (0.046); <0.001 |
| FW | -0.3 (0.04); <0.001 | -0.24 (0.041); <0.001 | -0.3 (0.049); <0.001 | -0.19 (0.05); <0.001 | -0.32 (0.051); <0.001 | -0.21 (0.053); <0.001 | -0.26 (0.048); <0.001 | -0.16 (0.051); 0.0015 |
| FA | 0.16 (0.041); <0.001 | 0.14 (0.041); <0.001 | 0.12 (0.042); 0.0041 | 0.11 (0.043); 0.0086 | 0.13 (0.045); 0.003 | 0.11 (0.045); 0.019 | 0.12 (0.042); 0.0047 | 0.094 (0.043); 0.031 |
| PSMD | -0.23 (0.042); <0.001 | -0.17 (0.042); <0.001 | -0.18 (0.047); <0.001 | -0.1 (0.047); 0.036 | -0.19 (0.048); <0.001 | -0.12 (0.049); 0.014 | -0.12 (0.046); 0.0069 | -0.063 (0.047); 0.18 |
| ALPS | 0.07 (0.042); 0.095 | 0.035 (0.042); 0.41 | 0.045 (0.044); 0.3 | -0.013 (0.044); 0.76 | 0.047 (0.045); 0.3 | 0.0043 (0.046); 0.92 | 0.0033 (0.042); 0.94 | -0.032 (0.044); 0.46 |

Values indicate linear regression statistics: beta (SE); p. Model M1 corresponds to the univariate association model.

FW: free water content; FA: fractional anisotropy; PSMD: peak width of skeletonized mean diffusivity (PSMD); ALPS: diffusion tensor image analysis along the perivascular space index; TCV: total cranial volume; EF: executive function; EM: episodic memory; Δ: annual change.

**Supplemental** **Table S3** **Bayesian Model Averaging** with baseline cognitive outcomes in non-demented participants

|  | Executive Function | | | Episodic Memory | | |  |
| --- | --- | --- | --- | --- | --- | --- | --- |
|  | Posterior probability | Estimate | SE | Posterior probability | Estimate | SE | |
| FW | 91.5 | -0.197 | 0.044 | 13.6 | -0.011 | 0.045 | |
| FA | 0.9 | 0.000 | 0.043 | 3.6 | 0.001 | 0.038 | |
| PSMD | 9.5 | -0.012 | 0.044 | 10.9 | -0.007 | 0.039 | |
| WMH | 42.3 | -0.049 | 0.043 | 100 | -0.193 | 0.038 | |
| ALPS | 14.9 | 0.011 | 0.039 | 3 | 0.001 | 0.036 | |
| Hippocampus | 100 | 0.243 | 0.037 | 100 | 0.462 | 0.035 | |
| Age | 2.7 | -0.001 | 0.044 | 0 | 0.000 | NA | |
| Sex | 100 | 0.210 | 0.042 | 100 | 0.246 | 0.036 | |
| Education | 100 | 0.438 | 0.037 | 100 | 0.240 | 0.035 | |
| Hypertension | 2.7 | -0.001 | 0.037 | 0 | 0.000 | NA | |
| Diabetes | 64.9 | -0.066 | 0.037 | 0 | 0.000 | NA | |
| TCV | 65 | 0.086 | 0.048 | 9.1 | -0.007 | 0.046 | |

FW: free water content; FA: fractional anisotropy; PSMD: peak width of skeletonized mean diffusivity (PSMD); ALPS: diffusion tensor image analysis along the perivascular space index; TCV: total cranial volume. A standard error (SE) reported as NA indicates that the variable was not selected in any of the models

**Supplemental** **Table S4** **Bayesian Model Averaging** with longitudinal cognitive outcomes in non-demented participants

|  | Annual change in Executive Function | | | Annual change in Episodic Memory | | |  |
| --- | --- | --- | --- | --- | --- | --- | --- |
|  | Posterior probability | Estimate | SE | Posterior probability | Estimate | SE | |
| FW | 66.4 | -0.105 | 0.055 | 3.5 | -0.002 | 0.053 | |
| FA | 3.4 | 0.002 | 0.042 | 0 | 0.000 | NA | |
| PSMD | 5 | 0.001 | 0.053 | 0 | 0.000 | NA | |
| WMH | 96.6 | -0.187 | 0.045 | 100 | -0.213 | 0.043 | |
| ALPS | 2.4 | -0.001 | 0.042 | 3.7 | -0.001 | 0.041 | |
| Hippocampus | 100 | 0.359 | 0.040 | 100 | 0.321 | 0.041 | |
| Age | 20.7 | 0.022 | 0.048 | 0 | 0.000 | NA | |
| Sex | 100 | 0.145 | 0.039 | 79.4 | 0.097 | 0.042 | |
| Education | 96.2 | 0.130 | 0.039 | 6.1 | 0.003 | 0.040 | |
| Hypertension | 0 | 0.000 | NA | 3.6 | 0.001 | 0.041 | |
| Diabetes | 0 | 0.000 | NA | 5.9 | 0.003 | 0.041 | |
| TCV | 0 | 0.000 | NA | 10.8 | -0.008 | 0.047 | |

FW: free water content; FA: fractional anisotropy; PSMD: peak width of skeletonized mean diffusivity (PSMD); ALPS: diffusion tensor image analysis along the perivascular space index; TCV: total cranial volume. A standard error (SE) reported as NA indicates that the variable was not selected in any of the models


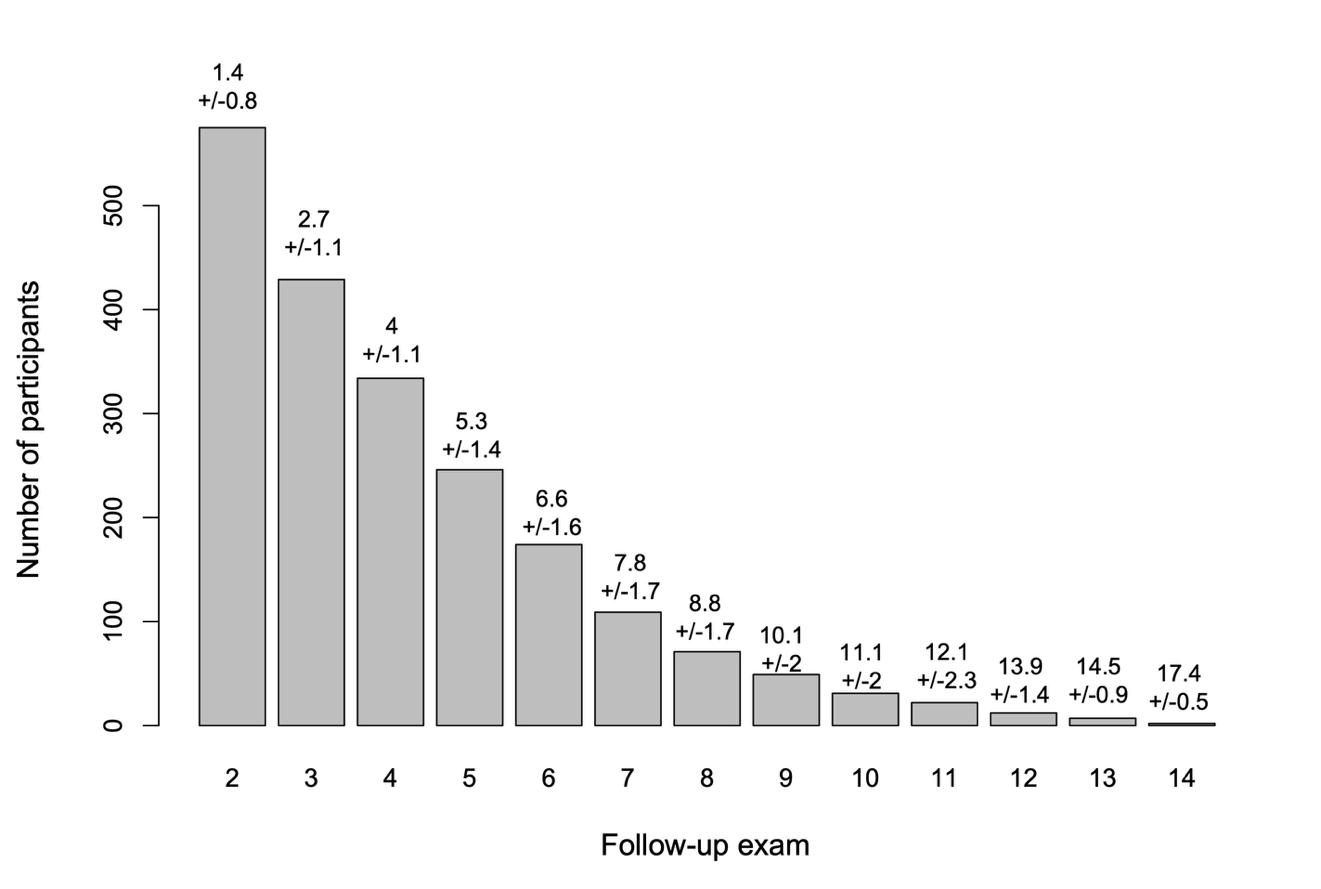
**Supplemental** **Figure S1**: Number of participants at each follow-up clinical examination and time difference (mean, +/- standard deviation in years) between baseline and the corresponding follow-up exam

**Supplemental** **Figure S2** Scatterplot illustrating the relationship between baseline executive function (EF) and its annual rate of change. Each point represents an individual participant, with the fitted regression line (blue) and 95% confidence interval (shaded area) shown. Higher baseline EF was associated with slower decline over time.


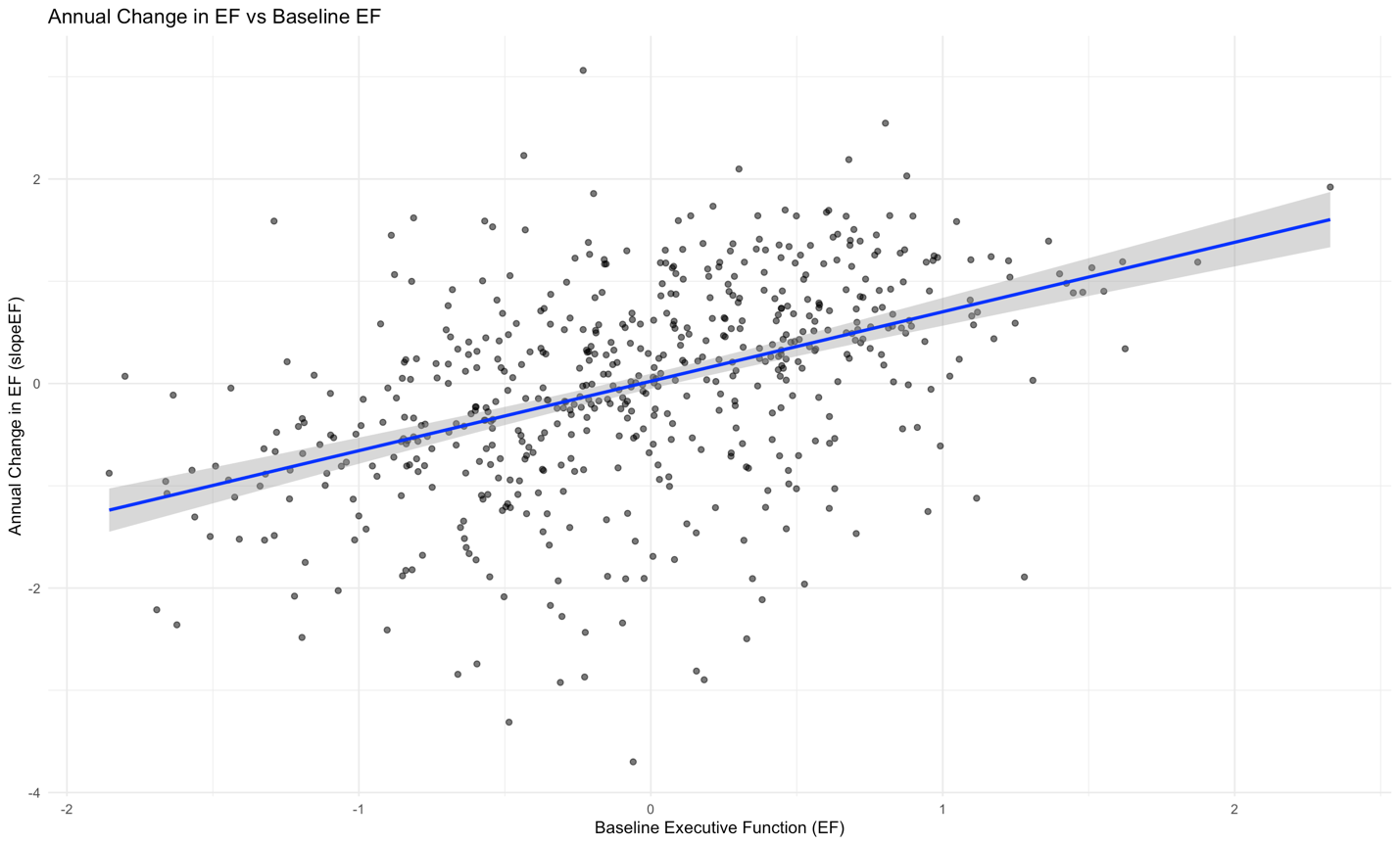


**Supplemental** **Figure S3** Scatterplot illustrating the relationship between baseline episodic memory (EM) and its annual rate of change. Each point represents an individual participant, with the fitted regression line (red) and 95% confidence interval (shaded area) shown. Higher baseline EM was associated with slower decline over time.


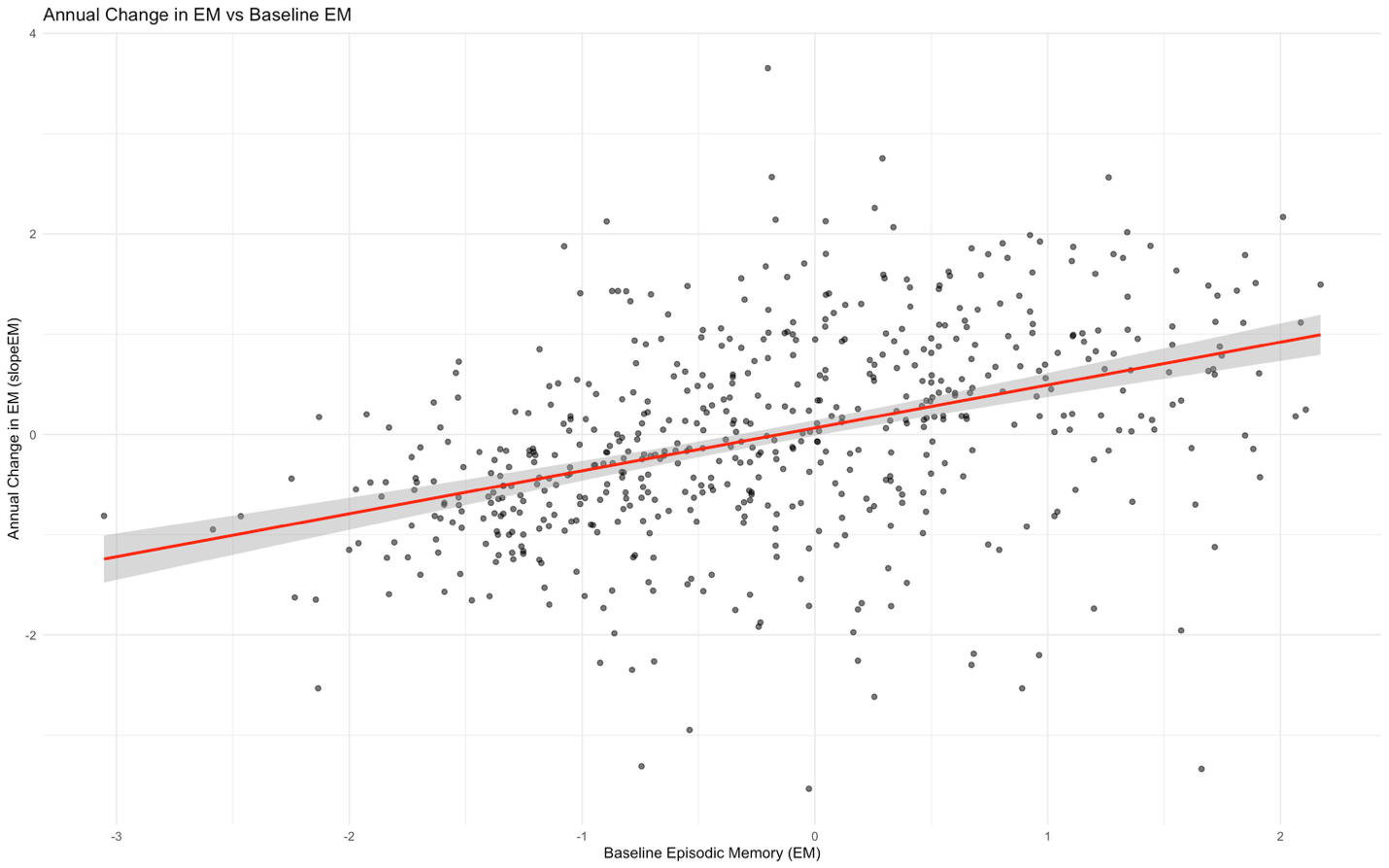


**Supplemental** **Figure S4 Bayesian Model Averaging posterior mean and posterior inclusion probability** in non-demented participants

Summary of **Bayesian Model Averaging models on baseline executive function (A) and episodic memory (**B) and change in **executive function (C) and episodic memory (**D). Number next to orange bar indicates the marginal inclusion probability of the variable (see Methods). FW: free water content; FA: fractional anisotropy; PSMD: peak width of skeletonized mean diffusivity (PSMD); ALPS: diffusion tensor image analysis along the perivascular space index; TCV: total cranial volume.

**Supplemental References**

1. DeCarli C, Fletcher E, Ramey V, Harvey D, Jagust WJ. Anatomical mapping of white matter hyperintensities (WMH): exploring the relationships between periventricular WMH, deep WMH, and total WMH burden. Stroke 2005;36:50-55.

2. Kochunov P, Lancaster JL, Thompson P, et al. Regional spatial normalization: toward an optimal target. J Comput Assist Tomogr 2001;25:805-816.

3. Jenkinson M, Beckmann CF, Behrens TE, Woolrich MW, Smith SM. Fsl. NeuroImage 2012;62:782-790.

4. Pierpaoli C, Basser PJ. Toward a quantitative assessment of diffusion anisotropy. Magn Reson Med 1996;36:893-906.

5. Pasternak O, Sochen N, Gur Y, Intrator N, Assaf Y. Free water elimination and mapping from diffusion MRI. Magn Reson Med 2009;62:717-730.

6. Smith SM, Kindlmann G, Jbabdi S. Chapter 10 - Cross-Subject Comparison of Local Diffusion MRI Parameters. In: Johansen-Berg H, Behrens TEJ, eds. Diffusion MRI (Second Edition). San Diego: Academic Press, 2014: 209-239.

7. Baykara E, Gesierich B, Adam R, et al. A Novel Imaging Marker for Small Vessel Disease Based on Skeletonization of White Matter Tracts and Diffusion Histograms. Ann Neurol 2016;80:581-592.

8. Smith SM, Jenkinson M, Johansen-Berg H, et al. Tract-based spatial statistics: voxelwise analysis of multi-subject diffusion data. NeuroImage 2006;31:1487-1505.

9. Johnson WE, Li C, Rabinovic A. Adjusting batch effects in microarray expression data using empirical Bayes methods. Biostatistics 2007;8:118-127.

10. Fortin JP, Parker D, Tunc B, et al. Harmonization of multi-site diffusion tensor imaging data. NeuroImage 2017;161:149-170.

11. Fortin JP, Cullen N, Sheline YI, et al. Harmonization of cortical thickness measurements across scanners and sites. Neuroimage 2018;167:104-120.

12. Pomponio R, Erus G, Habes M, et al. Harmonization of large MRI datasets for the analysis of brain imaging patterns throughout the lifespan. Neuroimage 2020;208:116450.
